# The thematic series on the psychosocial and mental health impacts of terrorism and collective violence: narrative review of common themes from diverse events, places and research methods

**DOI:** 10.1192/bjo.2025.10061

**Published:** 2025-08-01

**Authors:** Richard Williams, Caroline Bell, Kenneth R. Kaufman

**Affiliations:** Welsh Institute for Health and Social Care, University of South Wales, Pontypridd, UK; Department of Psychological Medicine, University of Otago, Christchurch, New Zealand; Department of Psychiatry, Rutgers Robert Wood Johnson Medical School, New Brunswick, New Jersey, USA; Department of Psychological Medicine, Institute of Psychology, Psychiatry and Neuroscience, King’s College London, London, UK; Department of Psychiatry, University of Oxford, Oxford, UK

**Keywords:** Terrorism, collective violence, research methods, distress and/or disorders, short- and longer-term impacts on families and responders

## Abstract

Thematic series were introduced to *BJPsych Open* by the current Editor-in-Chief to address key topics in psychiatry and mental health, specifically considering the impact on the global burden of diseases with associated treatments, outcomes, policy and research priorities. The increasing submission to *BJPsych Open* of articles about the psychosocial and mental health impacts of terrorism and collective violence naturally led to this thematic series. This paper introduces the journal’s series of published papers about terrorism and collective violence. While we identify the topics covered by the series and hope to generate conversation, this paper does not report a systematic review of the series. The thematic series consists of 13 articles; 9 were open submissions and 4 were commissioned. They include this review, an editorial concerning research methods and 11 papers reporting how people have responded to terrorist and violent incidents in 4 countries. Including this review, one paper was published in 2020, three in 2022, two in 2023, five in 2024 and two in 2025. The commissioned papers were added to broaden coverage of the Utøya attack on young people in Norway, and the shootings in Christchurch, New Zealand in 2019. Our intention was to enable the papers on these two incidents to sit alongside papers already submitted about them and the Manchester Arena bombing as well as articles about attacks in Germany. We begin by introducing the papers and comment in the discussion on a series of topics that we have selected as prominent in the series.

The papers in this thematic series derive from a diversity of research approaches including qualitative, quantitative and mixed methods, use of a digital population research cohort, and systematic review. While the topics on which we comment in the discussion are not systematically derived, patterns do emerge from this series; they are supported by many other contemporary papers of which we can cite but a tiny selection. The topics we highlight are germane to creating and delivering high-quality care for patients and responders. They include the terminology for describing short- and medium-term impacts, the duration of impacts into the longer-term, and impacts on families and responders to incidents. The series affords sight of possible negative prognostic factors including the impacts of shame and guilt and the presence of secondary stressors as well as the typically more positive impacts of social support. We contrast the different findings from survey techniques and clinical interviews that resonate with findings from research on healthcarers conducted during the COVID-19 pandemic.

The series opens with a paper that reports surveys of the mental health of people in Germany in the early weeks after the start of the Russo-Ukrainian War.^1^ It includes reports of two assessments of a subsample of 4441 people from the German population-based cohort for digital health research showing that symptoms of anxiety measured by General Anxiety Disorder-7 (GAD-7) in the first weeks of war exceeded reactions during the strongest COVID-19 restrictions. Evidently, fear of the impact of war is associated with worse mental health and people who are not directly involved in violent events are affected as ripples of communicated fear spread.

Second, we highlight an editorial on lessons learned for research methods about psychosocial sequelae of terrorism from the team that conducted studies of survivors of the shootings at two mosques in Christchurch, New Zealand in 2019.^2^ It highlights some of the challenges of conducting research with minority populations. They include cultural and linguistic challenges, stigma and concerns about confidentiality. Alongside research using trauma-informed approaches, the authors emphasise the importance of a participatory approach and active community engagement. These actions build trust, identify and mitigate concerns, and ensure cultural appropriateness and that findings are of value to the community.

The third paper reports qualitative analysis of semi-structured interviews with a purposive sample of 18 people who, at assessment at 3 and 6 months after the bombing were mildly, moderately or more severely affected by their attendance at a pop concert in the Manchester Arena in 2017.^3^ The intention was to enhance understanding of the experiences of people directly affected but not physically injured and identify their opinions on their social support and recovery through thematic analysis of their semi-structured interviews that were conducted 30 months after the event. Among a wide number of themes, that paper comments on the longitudinal association between trauma-related shame and guilt and psychopathology.^3^ The fourth paper reports on trauma-related shame and guilt measured at 2.5 and 8.5 years in 347 survivors of the mass shooting on Utøya island in Norway.^4^ The authors say that shame and guilt may be prevalent in survivors of mass trauma several years after the event and and that shame, in particular, may play an important role in long-term mental health.

The fifth paper reports a study that examined the proactive approach to psychosocial care provided by municipal contact persons for the young people exposed to the attack at the Utøya youth camp.^5^ This longitudinal study found that survivors with a contact person early after the attack were less likely to receive care from mental health services or to have symptoms of anxiety or depression subsequently compared with survivors without a contact person. However, there was considerable variation in the perceived usefulness and duration of the follow-up. The authors note that, due to conflicting results, they could not reach a firm conclusion about the potential health and social benefits or disadvantages of the proactive interventions implemented. They recommend further research on this important topic.

The next two papers^6,7^ concern the people who survived the mosque attacks in Chistchurch, New Zealand in 2019; they report quantitative and qualitative findings. The quantitative paper reports high rates of mental disorders in a sample of 189 participants of survivors and affected Muslim community members 11–32 months after the attacks.^6^ The authors confirm findings from previous studies after terror attacks and highlight that being bereaved or directly experiencing such horrific events, whether injured or not, is associated with adverse mental health outcomes. They emphasise the importance of including everyone in screening and access to support. The qualitative study provides further detail about people’s experiences.^7^ Alongside the impact of distress and secondary stressors, and the importance of connectedness, the authors note the centrality of the Islamic faith in the experiences of this minority community. The authors observe that post-traumatic growth was not solely related to religion. They emphasise the need for therapeutic approaches to include a focus on enhancing positive religious coping and maximising the potential for spiritual growth as a resource.

The eighth paper returns to the Manchester Arena bombing to report research on informal psychosocial care that is provided by families, friends, peer groups and wider social networks. It used thematic analysis of semi-structured interviews with survivors to derive recommendations about social validation and how interventions for survivors and their social networks might be facilitated.^8^ The ninth paper is also about the impact of the bombing in Manchester. It used mixed methods to explore questions about the experiences of distress reported by survivors, how secondary stressors influenced their recoveries, and the part played by social support in survivors’ trajectories of recovery. The paper shows the power of bringing together longitudinal quantitative research and qualitative findings with information about the progress and recovery or otherwise of people more than three years after the attack.^9^

Key to improving care offered to people affected by collective violence is understanding the impacts of terror attacks on the psychosocial needs and mental health of emergency responders over an extended time frame. The 10th paper reports a systematic review of the impacts on responders that critiques 33 papers concerning 159 621 participants over times varying from 2 weeks to 13 years.^10^ The 11th paper is a short report on long-term paranoid ideation in female emergency personnel after a terror attack in Berlin, Germany.^11^ The authors of the latter opine that their results could argue for development of gender-specific interventions before and after deployments of staff on operations in emergencies.

Our 12th and last paper analyses public violence in the context of other contemporary societal incidents.^12^ A critical issue is how use of social media precipitated national riots in the UK in 2024

after the death of three children in a knife attack. Misinformation about the suspect’s heritage and religion was spread by social media, which was then used to incite violence and racism resulting in damage to property, terror and injuries.^12^

Nine papers in the thematic series cover events that have in common extreme violence thought at the time to have been perpetrated by lone actors against unsuspecting collective groups of the public. We recognise that there are other patterns, including state-sponsored terrorism and terrorist incidents conducted on behalf of groups.

## Discussion

We draw attention to a selection of topics that arise from the thematic series but there are, of course, others.

### Distress and disorders

We think an important topic is how terminology to describe impacts is still evolving to reflect broad constructs and developments in bringing together social, psychological and psychiatric considerations. An important question is how we construe the psychosocial and mental health impacts of violence and terrorism. When one of us, R.W., was first engaged in practice in this area more than 40 years ago, there were uncertainties about how we should apply diagnoses to people’s reactions to terrorism and post-traumatic stress disorder (PTSD) was a new diagnostic possibility. Subsequently, acute stress disorder (ASD) was conceived to describe the reactions of people that resembled PTSD in the first 4 weeks after incidents as a way of identifying people at higher risk of developing PTSD.

However, we note that the authors of many of the papers in the thematic series describe people’s early reactions more simply as their suffering ‘distress’.^3–5,7–9^ Thus, a number of the papers in this series describe participants as distressed rather than applying diagnoses in the short-term. By contrast, Karam et al espouse the importance of practitioners recognising the presence of symptoms of ASD, and particularly their intensity.^13^

This takes us to a related matter, which is raised by one of the papers in the series, concerning how we should measure the impacts on people of incidents that include terrorism.^10^ Some researchers report epidemiological findings from validated and commonly used measures of people’s symptoms. Often, they employ self-report methods and use cutoffs that researchers have calculated in previous research in other circumstances to offer estimates of caseness. However, what is becoming clearer is that using self-report survey techniques with convenience samples that are poorly defined tends to provide estimates of the prevalence of common mental disorders and PTSD that are notably higher than findings from standardised clinical interviews. This matter was brought to prominence by surveys conducted during the COVID-19 pandemic.^14^ In the series, the authors of the systematic review of 33 papers draw attention to this matter in relation to terrorism and the authors opine that when ‘questionnaire results … [are] utilised in future research, this should always be described in the limitations’.[^10^ p.10] Our opinion is that this is a reason for being cautious about ascribing diagnoses in the early days and weeks after incidents to people affected by terrorism and collective violence when self-report methods are used in surveys. We qualify the recommendation from Karam et al^13^ by suggesting that researchers should take into account how the data they interpret were collected when deciding on whether they are confident to offer diagnoses for people whom they have not examined. Certainly, these observations indicate the need for further research into how best to compare and integrate the results of self-report questionnaire and clinical interview studies.

Similar challenges of terminology and measurement apply in the medium- and longer-terms as well as the short-term. In the past, we anticipated that distress would ameliorate relatively quickly in the early aftermath before diagnoses of common mental disorders and PTSD became appropriate, but the findings from the papers about the Manchester Arena bombing and the Utøya shooting imply that a significant proportion of uninjured people may continue to be distressed over many months. Whether they do or do not appears to turn, to an extent at least, on the intensity of their early suffering, their previous childhood experiences, how they are assisted, their current relationships and their levels of affluence. Consequently, the notion of active monitoring over time has gained sway.^15^ The paper regarding psychosocial follow-up after the Utøya shootings evaluates a model of active monitoring of people involved in incidents. While its authors were not able to reach a firm conclusion about proactive follow-up, there were clear indications from the Manchester Arena studies that relationships shared with other people affected by the same incident may prove mutually beneficial in line with science about the social cure.^8^

All these circumstances are reminiscent of the literature on the impacts of COVID-19 and other disasters.^16,17^ Immediate surveys may have some merit, but our opinion is that using mixed-method, longitudinal studies is key to understanding symptom progression, implicated factors and causality. The papers from Norway, New Zealand and the UK illustrate this matter.

### Impacts on families

The impacts on families affected by terror attacks are consistently noted in many papers. A number of the studies included young people among the participants.^3–5,7–9^ The research on the Manchester Arena bombing showed that the quality of close relationships is pivotal to long-term outcomes.^9^

As previously reported, deaths of close relatives in terror attacks are associated with mental disorders, particularly PTSD and depression.^6^ But, the families of survivors also report the toll on them from witnessing the suffering and enduring distress experienced by their loved ones.^3,7^ Stancombe et al report that some parents described high levels of stress and feelings of guilt in exposing their children to the Manchester bombing, feeling unable to provide adequate care themselves, and stress associated with not being able to access support they thought appropriate.^3^

What is not addressed in the first 11 papers is what occurs when parents bring children to riots or coach them to participate. The 12th paper in our series considers that

community cohesion and race relations seemed to crash [in 2024] to levels not seen for many years in Britain. What was even more troubling was that rioting parents took their children to witness the violence, harmful to them in so many ways. In some instances, the children were coached to participate and throw missiles at the police, which led to two 12 year olds being charged for violent disorder.^12^

Conversely, several papers in our series highlight the important roles families play in providing support after terror attacks, echoing previous findings that they are very often the preferred avenue of care.^3,6–9^ However, we should not assume that helpful support occurs naturally, and survivors can be constrained from sharing feelings with their families if the latter are perceived as being unable or less inclined to understand their experiences.^3,8^ This could potentially exacerbate distress and prolong recovery. These authors suggest services should facilitate processes that ensure that this support is constructive by increasing families’ understanding of survivors’ experiences and intra-familial communication.^9^

### Longer-term impacts

Disasters and terrorism have negative impacts on mental health with increased rates of symptoms of mental disorders. However, relatively less is known about longer-term as compared with short-term impacts. Several papers in this thematic series address this gap, examining outcomes 2 to 13 years post-incident.^5–7,9–11^

Long-lasting impacts on survivors and emergency responders have been reported consistently and three papers in this series make this point.^9–11^ Oppo et al report a qualitative systematic review and meta-analyses of 10 papers on studies of adult participants. They identified four symptom trajectories in 64% of studies by latent growth modelling: *low-stable* (the modal response with pooled prevalence of 58%), *high-stable* (7%), *decreasing* (13%) and *delayed-worsening* (8%); they also found a fifth trajectory of *moderately stable* (19%) in 50% of the studies.^18^ A paper in our series explores the trajectories of distress and recovery; it found three broad modal patterns of responding: low symptom levels and short-term distress (the largest group of people), more persistent distress and slower recovery (a substantial minority) and high stress and deterioration (in a smaller but significant minority).^9^ Thus, despite variations in the detail, terms in which the trajectories are described and measured, and number of trajectories, there appears to be broad consistency between the reports from Stancombe et al,^9^ Oppo et al^18^ and Bryant et al^19^ about how people respond over time to disasters and collective violence in a variety of different contexts.

Several papers in this issue explore some of the potential drivers of these patterns of responding. Confirming previous findings,^20,21^ the presence of physical symptoms such as changes in sleep and appetite, somatic concerns and inability to perform everyday activities were reported by people with more enduring functional impairment.^3^ The opinion of Stensland et al is that their ‘findings indicate that survivors’ early pain and related somatic symptoms strongly and consistently predict later psychopathology.’^21^ As identified, shame following a terror attack was explored by two of the studies we highlight.^3,4^ Both report that experiences of shame are prevalent years after the event with significant associations between shame and mental health outcomes.^3,4^ These findings suggest that somatic reactions and shame might serve as markers of risk or enduring problems. They may be important symptoms to include in research and are potential targets for intervention. They may also help us to differentiate people who are distressed from those who may progress to developing disorders.

Secondary stressors^22^ are discernable in a number of the papers whether or not they are described using that terminology. Their deleterious impacts on recovery are explored in several papers.^1–3,7–9,12^ They included family concerns, work stress, media involvement and barriers to or lack of healthcare and support. Interestingly, while social support is typically protective, one paper also reports that inappropriate or invalidating responses from families, friends and service providers can themselves act as secondary stressors.^3^ Thus, there is a component of risk concerning the nature and quality of social support that people are offered.

**Fig. 1:**
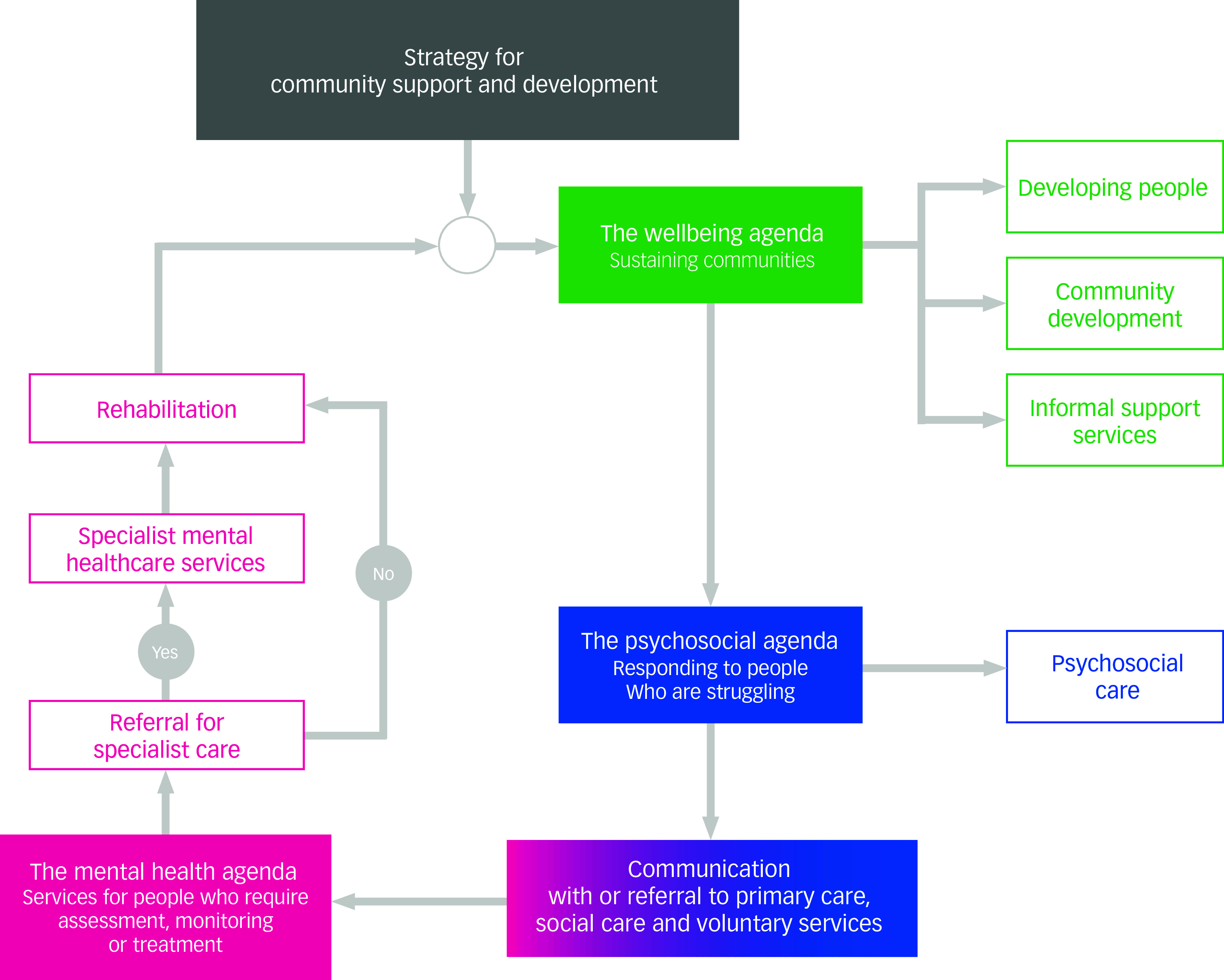
A strategic approach to responding to the needs of people affected by terrorist incidents (reproduced with permission from R Williams and V Kemp, 2020 – all rights reserved).

These findings argue for creating a clear but flexible framework for interventions to support the likely different levels of need after a terror attack (i.e. supporting the well-being of everyone affected, providing psychosocial interventions for distressed people who are struggling, and assessing and treating people who have mental disorders) [^16^ identifies this framework]. Fig. 1 offers a diagramatic representation of this approach. A consistent finding is that the impacts are often long-lasting, and it is now clear that support services should be available for a number of years after incidents.

### Responders

Two papers in this thematic series explore the impacts of terrorist attacks on responders.^10,11^ The main findings of the first showed that prevalence rates of PTSD, major depression and specific anxiety disorders were inconsistent and that the spectrum of symptoms is much broader than those of PTSD and other diagnoses.^10^ As noted earlier, this raises possibilities that self-report surveys might risk confusing distress with diagnosable disorders; this might be one reason why prevalence rates reported in studies using self-reported questionnaires are so much higher than when people are assessed in clinical interviews.^14^

We make two further points. First, our experiences of the COVID-19 pandemic drew our attention to the needs of healthcare staff and, indeed, all responders, but it is also clear that the needs of staff had gradually been becoming more apparent and better recognised over a much longer time frame.^23,24^ The needs of carers and responders have not been resolved as the prevalence of COVID-19 has slowly but sporadically reduced across many countries’ populations. Thus, it is not solely emergency responders who require improved care and support, but all healthcarers.^24^

Second, what form should responses to the needs of staff take? We are not drawn to specific mental health treatments as the foundation of society’s initial responses to the experiences and needs of responders, though some of them may require mental health care as time progresses. The discussion in the paper by Wesemann et al is helpful.^10^ It includes the statement that institutional peer support was found to be effective after the 9/11 World Trade Center attacks and is probably the most appropriate way to deal with such huge incidents.^25^ Several papers in our series suggest that survivors are helped by social support though the papers by Stene et al,^5^ and Stancome et al^3^ indicate that the researched position on effectiveness may be complicated and turn on other interacting factors (such as past experiences and affluence).^5^ Others papers, including many that are published elsewhere, indicate that staff of healthcare services benefit from peer support as incidents unfold, in the immediate aftermath and also in the longer-term.^10,24–26^ Thus, ‘Psychosocial Care’ in Fig. 1 includes social support for survivors and peer support for responders and other staff.

### Lessons for designing and delivering services in the future

The papers included in this series illustrate knowledge that should influence how we design, deliver and sustain services and supports for people affected by collective violence. Some papers explore events and people’s perceptions of them through qualitative approaches. Others use quantitative, epidemiological research techniques and/or analysis of large data sets. Many show the importance of statistical analysis and the growing influence of longitudinal statistical approaches. Some of the papers included in the series reflect the themes of prevention through, for example, social support systems and minimising secondary stressors. Bhui et al recommend a range of other approaches including the importance of addressing the powerful influences of social media.^12^

In conclusion, we think that further development is needed on many fronts including how best to define, prevent, determine, monitor and respond to the impacts of violent incidents on survivors so that our interventions become synergistic and promote effective person-centred, culturally appropriate care through alliances between survivors of past incidents, people at risk, practitioners, researchers, policymakers and the responsible authorities.

## Data Availability

Data availability is not applicable to this article as no new data were created or analysed in this study.

## References

[1] Gottschick C , Diexer S , Massag J , Klee B , Broda A , Purschke O , et al. Mental health in Germany in the first weeks of the Russo-Ukrainian war. BJPsych Open 2023; 9: e66. 37057843 10.1192/bjo.2023.21 PMC10134205

[2] Sulaiman-Hill R , Porter R , Schluter P , Beaglehole B , Dean S , Tanveer S , et al. Research following trauma in minority ethnic and faith communities: lessons from a study of the psychosocial sequelae of the Christchurch mosque terror attacks. BJPsych Open 2024; 10: e27. 38205604 10.1192/bjo.2023.641 PMC10790214

[3] Stancombe J , Williams R , Drury J , Collins H , Lagan L , Barrett A , et al. People’s experiences of distress and psychosocial care following a terrorist attack: interviews with survivors of the Manchester Arena bombing in 2017. BJPsych Open 2022; 8: e41. 35109959 10.1192/bjo.2022.2 PMC8867861

[4] Glad KA , Aakvaag HF , Wentzel-Larsen T , Dyb G , Thoresen S. What will others think of me? The longitudinal association between trauma-related shame and guilt and psychopathology after a terror attack. BJPsych Open 2024; 10: e30. 38205599 10.1192/bjo.2023.624 PMC10790223

[5] Stene LE , Glad KA , Stensland SØ , Nilsen LG , Dyb G. Proactive psychosocial follow-up of youth exposed to a terrorist attack: a longitudinal study linking interviews and register-based data. BJPsych Open 2025; 11: e48. 40071476 10.1192/bjo.2024.838 PMC12001956

[6] Bell C , Sulaiman-Hill R , Tanveer S , Porter R , Dean S , Schluter PJ , et al. Factors associated with mental health outcomes in a Muslim community following the Christchurch terrorist attack. BJPsych Open 2024; 10: e209. 39534916 10.1192/bjo.2024.774 PMC11698172

[7] Dean S , Eggleston K , Ali F , Thaufeeg Z , Wells H , Zarifeh J, et al. ‘I can feel sad about it and I can worry, but inside, I know everything happens for a reason’: personal experiences in the aftermath of the March 15 Christchurch mosque attacks. BJPsych Open 2024; 10: e176. 39391924 10.1192/bjo.2024.791 PMC11536286

[8] Drury J , Stancombe J , Williams R , Collins H , Lagan L , Barrett A , et al. Survivors’ experiences of informal social support in coping and recovering after the 2017 Manchester Arena bombing. BJPsych Open 2022; 8: e124. 35781122 10.1192/bjo.2022.528 PMC9301776

[9] Stancombe J , Williams R , Drury J , Hussey L , Gittins M , Barrett A , et al. Trajectories of distress and recovery, secondary stressors and social cure processes in people who used the resilience hub after the Manchester Arena bombing. BJPsych Open 2023; 9: e143. 37550867 10.1192/bjo.2023.527 PMC10594089

[10] Wesemann U , Applewhite B , Himmerich H. Investigating the impact of terrorist attacks on the mental health of emergency responders: systematic review. BJPsych Open 2022; 8: e107. 35656574 10.1192/bjo.2022.69 PMC9230690

[11] Wesemann U , Mahnke M , Polk S , Willmund G. Long-term effects of the terror attack in Berlin in 2016 on paranoid ideation in female emergency personel. BJPsych Open 2020; 6: e79. 32741399 10.1192/bjo.2020.57 PMC7453799

[12] Bhui K , Roberts D , Lashley M , Jones E , Kaufman KR. Extremism, racism and riots: exploring the political, social and cultural determinants of poor mental health. BJPsych Open 2024; 10: e224. 39635737 10.1192/bjo.2024.830 PMC11698177

[13] Karam EG , Al Barathie J , Dimassi H , Mascayano F , Slim A , Karam A , et al. Unveiling the neglected role of the intensity of acute stress disorder in the prediction of full- and sub-threshold posttraumatic stress disorder: looking beyond the diagnosis. Soc Psychiatry Psychiatr Epidemiol 2025; 60: 1125–33. 39738651 10.1007/s00127-024-02805-z PMC12119768

[14] Scott HR , Stevelink SAM , Gafoor R , Lamb D , Carr E , Bakolis I , et al. Prevalence of post-traumatic stress disorder and common mental disorders in healthcare workers in England during the COVID-19 pandemic: a two-phase cross-sectional study. Lancet Psychiatry 2023; 10: 40–9. 36502817 10.1016/S2215-0366(22)00375-3 PMC9731576

[15] National Institute for Health and Care Excellence. Post-Traumatic Stress Disorder NICE Guideline NG116. NICE, 2018. 31211536

[16] Murray E , Kaufman KR , Williams R. Let us do better: learning the lessons for recovery of healthcare professionals during and after COVID-19. BJPsychOpen 2021; 7: e151. 10.1192/bjo.2021.981 PMC8376907 34457351

[17] Bryant RA , Gibbs L , Gallagher HC , Pattison P , Lusher D , MacDougall C , et al. Longitudinal study of changing psychological outcomes following the Victorian Black Saturday bushfires. Aust NZ Psychiatry 2018; 52: 542–51. 10.1177/0004867417714337 28605987

[18] Oppo A , Forresi B , Barbieri A , Koenen KC. Trajectories of posttraumatic stress symptoms following collective violence: a systematic review and meta-analyses. J Trauma Stress 2024; 37: 837–49. 39176467 10.1002/jts.23090

[19] Bryant RA , Nickerson A , Creamer M , O’Donnell M , Forbes D , Galatzer-Levy I , et al. Trajectory of post-traumatic stress following traumatic injury: 6-year follow-up. BJPsych 2015; 206: 417–23. 25657356 10.1192/bjp.bp.114.145516

[20] Bugge I , Dyb G , Stensland S , Ekeberg Ø. , Wentzel-Larsen T , Diseth TH. Physical injury and somatic complaints: the mediating role of posttraumatic stress symptoms in young survivors of a terror attack. J Trauma Stress 2017; 30: 229–36. 28556275 10.1002/jts.22191

[21] Stensland SØ. , Thoresen S , Jensen T , Wentzel-Larsen T , Dyb G. Early pain and other somatic symptoms predict posttraumatic stress reactions in survivors of terrorist attacks; the longitudinal Utøya cohort study. J Trauma Stress 2020; 33: 1060–70. 32662140 10.1002/jts.22562

[22] Williams R , Ntontis E , Alfadhli K , Drury J , Amlôt R. A social model of secondary stressors in relation to disasters, major incidents and conflict: implications for practice. Int J Disast Risk Reduc 2021; 63: 102436.

[23] Williams R , Kemp V. Caring for healthcare practitioners. BJPsych Adv 2020; 26: 116–28.

[24] Oeppen RS , Melville CR , Brennan PA. The NHS should do more to prevent fatigue in healthcare staff. BMJ 2023; 383: 2676. 37967893 10.1136/bmj.p2676

[25] Dowling FG , Moynihan G , Genet B , Lewis J. A peer-based assistance program for officers with the New York City police department: report of the effects of Sep 11, 2001. Am J Psychiatry 2006; 163: 151–3. 16390904 10.1176/appi.ajp.163.1.151

[26] Williams R , Kemp V , Burgess J , Murray E , Stokes S , Wood A , et al. Practical psychosocial care for providers of pre-hospital care: a summary of the report ‘valuing staff, valuing patients’. Scand J Trauma Resusc Emerg Med 2023; 31: 77. 37946286 10.1186/s13049-023-01141-6 PMC10636848

